# NTRK2 Fusion driven pediatric glioblastoma: Identification of oncogenic Drivers via integrative Genome and transcriptome profiling

**DOI:** 10.1002/ccr3.3804

**Published:** 2021-02-10

**Authors:** Heidi M. Britton, Adrian B. Levine, Yaoqing Shen, Karen Mungall, Jonathan Serrano, Matija Snuderl, Erin Pleasance, Steven J. M. Jones, Janessa Laskin, Marco A. Marra, Shahrad R. Rassekh, Rebecca Deyell, Stephen Yip, Sylvia Cheng, Chris Dunham

**Affiliations:** ^1^ Department of Pathology and Laboratory Medicine University of British Columbia Vancouver Canada; ^2^ Canada's Michael Smith Genome Sciences Centre British Columbia Cancer Agency Vancouver Canada; ^3^ Division of Anatomic Pathology Children's and Women's Health Centre of British Columbia Vancouver BC Canada; ^4^ Division of Pediatric Hematology/Oncology British Columbia Children's Hospital and the University of British Columbia Vancouver BC Canada; ^5^ Department of Medical Oncology British Columbia Cancer Agency Vancouver BC Canada; ^6^ Department of Pathology New York University School of Medicine New York NY USA

## Abstract

This is the first report of a NACC2‐NTRK2 fusion in a histological glioblastoma. Oncogenomic analysis revealed this actionable fusion oncogene in a pediatric cerebellar glioblastoma, which would not have been identified through routine diagnostics, demonstrating the value of clinical genome profiling in cancer care.

## Graphical abstract

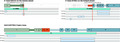

## 1 INTRODUCTION

Pediatric brain tumors represent the most common solid childhood cancer and the leading cause of death among childhood cancers, with an age‐adjusted incidence rate of 5.26 per 100,000 in children 0‐14 years of age.^1^ The most common tumor types are medulloblastoma and pilocytic astrocytoma, while pediatric glioblastoma is comparatively rare, accounting for only 2.5% of childhood brain tumors, of which only ~ 5% arise in the cerebellum.^2^, ^3^ Glioblastoma is associated with very poor outcomes, with a mean survival of approximately one year.^4^

The rapid expansion of oncogenomics has led to incorporation of molecular markers into the 2016 WHO CNS tumor classification that describes distinct subgroups such as the H3F3A K27M‐mutant diffuse midline glioma.^5^ These tumors are often seen in the pons, thalamus, and spinal cord but have also been reported in the cerebellum, among other locations.^6^ Subsequent molecular analysis of over 1,000 high‐grade pediatric gliomas gave further insight into the heterogeneity of H3/IDH‐wild‐type tumors, illustrating their divisibility into several prognostically distinct molecularly defined subgroups.^7^

Oncogenic gene fusions are increasingly recognized as drivers in a wide range of cancer types, with estimates that 20% of cancer morbidity may be attributable to fusion‐driven malignancies.^8^ With the recent success of drugs like crizotinib,^9^ gene fusions are generating significant interest as an avenue for targeted personalized oncology. However, traditional methods of fusion identification, such as FISH and RT‐PCR, are inadequate for the large‐scale identification of novel fusions, highlighting the importance of clinical sequencing and bioinformatics.^10^

The Neurotrophic Receptor Tyrosine Kinase genes *NTRK1‐3* encode for the tropomyosin receptor kinase (TRK) proteins A‐C, a family of receptor tyrosine kinases that are involved in the maturation of the nervous system, through their roles in neuronal differentiation, proliferation, and migration, as well as synapse development.^11^ The primary TRKB ligand is brain‐derived neurotrophic factor, the binding of which leads to receptor dimerization, auto‐phosphorylation, and downstream activation of several potential pathways, including PI3K‐AKT and RAS‐MAPK. *NTRK* fusions have been implicated in numerous cancer types, and recent phase 1/2 basket trials using the pan‐TRK inhibitor Larotrectinib in both children and adults have demonstrated positive results.^12^, ^13^ Activating *NTRK* fusions are only seen in 5% of pediatric high‐grade gliomas; however, half of these are in children under age three.^14^

## 2 CLINICAL HISTORY

An eleven‐month‐old girl presented with a three‐week history of head tilt to the right side, irritability, and developmental regression, and a one‐week history of daily emesis. She was no longer cruising and had significantly decreased motor activity. Her fontanels were open and an ultrasound showed dilated 3rd and 4th ventricles. A subsequent MRI revealed a large (40 × 57 × 47 mm) ring‐enhancing posterior fossa mass, causing significant obstructive hydrocephalus (see supplemental Figure S1) and multiple satellite lesions in the posterior fossa, measuring up to 16 mm. There was no evidence of spinal disease on imaging, and the CSF was free of malignant cells. Pathology reported a glioblastoma and cytogenetics demonstrated homozygous *CDKN2A* loss with no other driver mutations identified by immunohistochemistry. A postoperative MRI showed a remaining satellite nodule above the resection cavity, with the majority of the left cerebellar tumor resected. Family history was negative for other CNS tumors, and genetic testing for neurofibromatosis type I (NF1) was negative.

Primary treatment consisted of a left occipital craniotomy for subtotal resection of the tumor, which was complicated by significant blood loss and hemodynamic instability. This was followed by four cycles of induction chemotherapy with Vincristine, Carboplatin, and Temozolomide, per Head Start III regimen C.^15^ Carboplatin was reduced by 50% during cycle 4 due to bilateral high‐frequency hearing loss. She underwent subsequent consolidation chemotherapy with high‐dose Carboplatin and Thiotepa with autologous stem cell rescue. The disease was stable in the resection cavity, but a second lesion (19 x 8 x 8 mm) was identified in the vermis. A biopsy from the resection cavity was obtained during the second‐look surgery, six months after primary diagnosis, and submitted for genomic analysis.

Despite the identification of a *NTRK* gene fusion, the family decided against enrollment in an *NTRK* inhibitor clinical trial. Instead, the patient received a course of proton beam radiation therapy (total dose of 50.84 Gy), followed by a maintenance course of Temozolomide and Lomustine.

The patient is now three years old and at most recent follow‐up, over 2 years after initial diagnosis, is in disease remission with stable follow‐up imaging. She attends daycare and wears hearing aids for her hearing loss. Her motor, fine motor, and social development are excelling with a Lansky Play‐Performance score of 100.

## 3 RESULTS

### 3.1 Pathology

**Figure 1:**
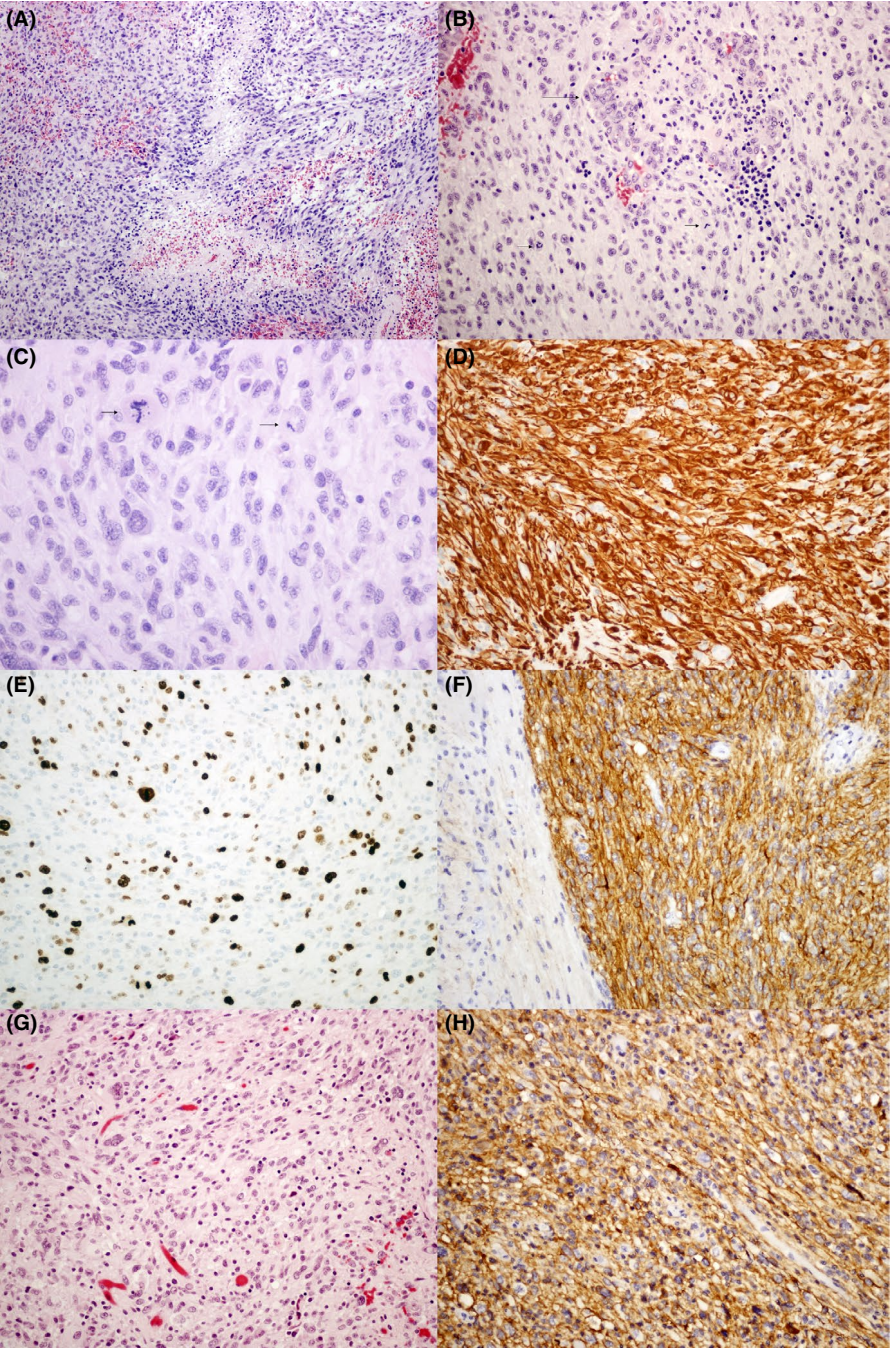
Histology from the original tumor (A‐F) and recurrence (G‐H). 1A) Pseudopalisading necrosis (H&E, 100x). 1B) Microvascular proliferation (H&E, 200x). 1c) Routine staining and high magnification reveal pleomorphic tumor astrocytes and an atypical mitotic figure (H&E, 400x). 1d) GFAP immunohistochemistry reveals strong reactivity in tumor cells (200x). 1E) The Ki67 proliferative index is high and estimated at 30%‐40% (200x). 1F) Immunoreactivity for PDL1 is strong in the tumor cells (right) and negative in the adjacent molecular layer of the cerebellar cortex (200x). 1G) Pleomorphic tumor astrocytes in the recurrent tumor (200x). 1H) PDL1 immunoreactivity remains strong in the tumor cells (200x)

Histopathology from the initial resection (Figure 1A‐F) showed a moderate to highly cellular tumor infiltrating cerebellar white matter and cortex, composed of highly pleomorphic tumor cells in a fibrillary background. Numerous mitotic figures were present alongside pseudopalisading necrosis and microvascular proliferation. There were no features suggestive of a low‐grade precursor.

Immunohistochemistry (IHC) showed strong GFAP positivity, with a high Ki‐67 index. IDH R132H, BRAF V600E, and H3F3A K27M were negative, p53 was focally positive, and ATRX and INI1 expression were retained. Cytogenetic analysis demonstrated homozygous deletion of *CDKN2A* on chromosome 9. Sections from the recurrent tumor (Figure 1G‐H) exhibited similar morphology, but with decreased mitotic activity and Ki‐67 staining, and the absence of necrosis or microvascular proliferation. Subsequent PD‐L1 IHC (SP142 antibody), confirmed the molecular testing results and demonstrated strong membranous positivity in both the initial tumor and recurrence.

### 3.2 Molecular

**Figure 2:**
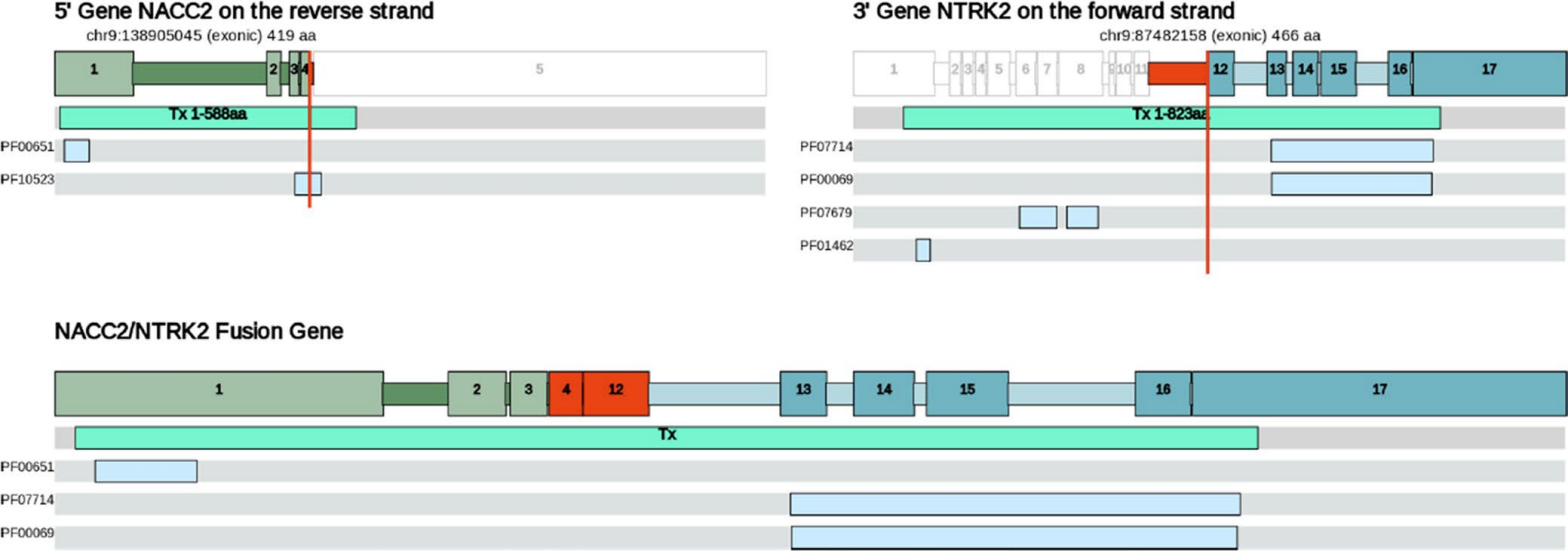
Putative fusion‐inversion event. The 5' (in the fusion) transcript NACC2‐001(ENST00000371753) from the gene NACC2(ENSG00000148411) on the reverse strand is drawn top left with its corresponding breakpoint at 9:138 905 045. The 3' (in the fusion) transcript NTRK2‐001(ENST00000323115) from the gene NTRK2(ENSG00000148053) on the forward strand is drawn top right with its corresponding breakpoint at 9:87 482 158. Exons are drawn to scale relative to other exons in the same drawing. Introns are scaled to make up approximately ¼ of the final drawing. Domain(s) featured in the above figure(s) are labeled by their various external identifiers as follows BEN_domain(PF10523); BTB_POZ(PF00651); Ig_I‐set(PF07679); LRR‐contain_N(PF01462); Prot_kinase_cat_dom(PF00069); Ser‐Thr/Tyr_kinase_cat_dom(PF07714)

Whole genome sequencing (WGS) data showed a homozygous deletion of both *CDKN2A* and *CDKN2B*, suggesting a dysregulated cell cycle. Both genomic and transcriptomic data showed a *NACC2:NTRK2* fusion, combining exons 1‐4 of *NACC2* with exons 15‐21 of *NTRK2* (Figure 2), with a one‐copy gain for each gene. Notably, a similar *NACC2:NTRK2* e4:e13 fusion has been reported in pilocytic astrocytoma.^6^ This *NACC2:NTRK2* e4:e15 fusion contained the kinase domain of *NTRK2*, while losing its ligand binding domain, suggesting constitutive activation of *NTRK2*. Therefore, NTRK2 inhibition was recommended as a therapeutic option.

Transcriptomic results revealed aberrant gene expression in several pathways. When compared to TCGA glioblastoma samples, a high percentile of expression was found in JAK1, JAK2, JAK3, and STAT1 in the JAK‐STAT pathway. In the IGF signaling pathway, IGF1 and IGF2 showed high expression levels, as did their receptor, IGF1R, and downstream substrates, IRS1 and IRS2. Several growth factors and their receptors, such as PDGFRB, NFGR, ALK, RET, and MET were also highly expressed.

Interestingly, this case showed a very high expression of CD274 (PD‐L1), which was later confirmed by IHC (Figure 1F and 1H). The mutation load of this case was low, at only 22 mutations. We did not find a high score of T cells as predicted by Cibersort,^16^ so the cause of the high expression of this gene remains unclear.

## 4 DISCUSSION

This clinical case report of a multifocal cerebellar glioblastoma in an eleven‐month‐old girl has several points of interest. Glioblastoma carries a very poor prognosis and is rare in children, particularly when localized to the cerebellum. As such, this patient was enrolled into the BC Cancer Personalized OncoGenomics (POG) trial, leading to the identification of potentially targetable mutations that would not have been discovered during routine workup. Ultimately, a targeted molecular approach to treatment was not pursued by the family, and the patient received standard treatment at our institution for infants with high‐grade brain tumors. Despite receiving standard treatment, the patient was doing very well at last follow‐up, over two years after initial diagnosis. This favorable preliminary outcome of glioblastoma is noteworthy and raises the possibility of an underlying favorable tumor biology.

In pediatric high‐grade gliomas, H3F3A mutations are the most common driver^7^; however, our case demonstrates a novel molecular profile. The major findings were an *NTRK* fusion and upregulation of PD‐L1 (as per transcriptomic data and IHC), both of which represent options for targeted therapy in clinical trials. The *NACC2:NTRK2* fusion, resulting from a complex inversion on chromosome 9, has been previously reported in a pilocytic astrocytoma.^17^ This fusion contains the kinase domain of *NTRK2* and a 5’ dimerization domain, likely leading to constitutive ligand‐independent activation of the protein product. Multiple *NTRK* inhibitors are in clinical trials, with some examples of successful use in brain tumors. For example, a heterogeneous glioblastoma in an adult, driven in part by a EML4‐NTRK3 fusion, was treated by Larotrectinib and exhibited a significant response in portions of the tumor.^18^

There is evidence that PD‐L1 is often expressed in glioblastomas, with reports of 61%‐88% of cases demonstrating some degree of staining.^19^, ^20^ Several checkpoint inhibitors have been approved, predominantly for use in melanoma and nonsmall cell lung cancer, and evidence from animal studies indicates that this treatment may impact glioblastomas.^21^ While the first phase‐3 clinical trial of Nivolumab in recurrent glioblastoma failed to prolong overall survival compared with Bevacizumab, several other clinical trials are in progress assessing checkpoint inhibitors, in combination with various other treatments.^22^ Despite the success that checkpoint inhibitors have had in treating non‐CNS tumors, there are several challenges to adapting immune‐onocologic strategies to glioblastomas, including the blood‐brain barrier, immunosuppressive tumor microenvironment, global immune dysfunction in these patients, and intratumoral heterogeneity.

The analysis by Mackay et al of over 1000 pediatric high‐grade gliomas identified a set of H3/IDH1‐wild‐type cases that had similar methylation profiles to pleomorphic xanthoastrocytoma (PXA) or low‐grade gliomas (LGG).^7^ These tumors were predominantly hemispheric, although some were seen in the cerebellum, and had improved survival, particularly in children under 12 months. Interestingly, our case demonstrates changes associated with both groups, as the PXA‐like tumors had frequent *CDKN2A/B* deletions, while a number of LGG‐like tumors had *NTRK* fusions. The possibility that our case belongs to one of these groups provides a reasonable biological explanation for the prolonged survival in an unequivocal histologic glioblastoma.

## 5 METHODS

For full methodology, please see the Supplemental Information. Histologic slides were prepared by standard techniques using the Ventana BenchMark XT Autostainer. PDL1 immunohistochemistry was performed using the primary clone SP142 as described previously.^23^ Molecular testing was done using frozen tissue and peripheral blood as a normal comparator. DNA and RNA sequencing was performed using the Illumina HiSeq platform v3, with additional targeted deep sequencing using the Ion Ampliseq oncogene panel platform and the IonTorrent PGM sequencing platform. Bioinformatic analysis was performed using methods previously described by our group.^24^ WGS identified somatic mutations, copy number changes, loss of heterozygosity, and structural variants. RNA sequencing confirmed the genomic findings from WGS and identified aberrant gene expression in comparison to the TCGA glioblastoma cohort. Additionally, RNA sequencing data from normal brain tissue from the Illumina Bodymap were used to calculate fold change of gene expression relative to normal.

## 6 ETHICS STATEMENT

A guardian of the patient provided written informed consent to participate, and this study was approved by the University of British Columbia Research Ethics Board as part of the Personalized Oncogenomics trial (NCT02155621) and represents part of the Personalized Oncogenomics trial NCT02155621.

## CONFLICT OF INTEREST

The authors declare no competing interests.

## AUTHOR CONTRIBUTIONS

HB and AL: wrote manuscript. HB: collected clinical information. YS: performed bioinformatic analysis. KM: provided structural variant data analysis and figures. JS and MS: performed methylation analysis. EP: performed bioinformatic analysis. SJ: reviewed sequencing and bioinformatics. JL: contributed to conception and design of this study. MM: reviewed sequencing and bioinformatics. RR and RD: provided patient treatment and clinical care. SY and CD: provided research supervision. SC: acted as clinical liaison and provided manuscript review. CD: provided pathologic analysis.

## Supporting information

Fig S1

Supplementary Material

## Data Availability

Data supporting the findings of this study are available from the corresponding author by request.
