# Supplementary material for: NTRK2 Fusion driven pediatric glioblastoma: Identification of oncogenic Drivers via integrative Genome and transcriptome profiling
Source: Clin Case Rep. 2021 Feb 10;9(3):1472–7. doi: 10.1002/ccr3.3804 (PMC7981675; doi:10.1002/ccr3.3804)

**SUPPLEMENTAL INFORMATION**

**Figure S1:** Pre-operative MRI. 1a) T1 axial with gadolinium reveals a large, ring enhancing tumor in the left cerebellar hemisphere. 1b) T2 axial demonstrates hydrocephalus and periventricular hyperintensity in keeping with transependymal CSF flow.


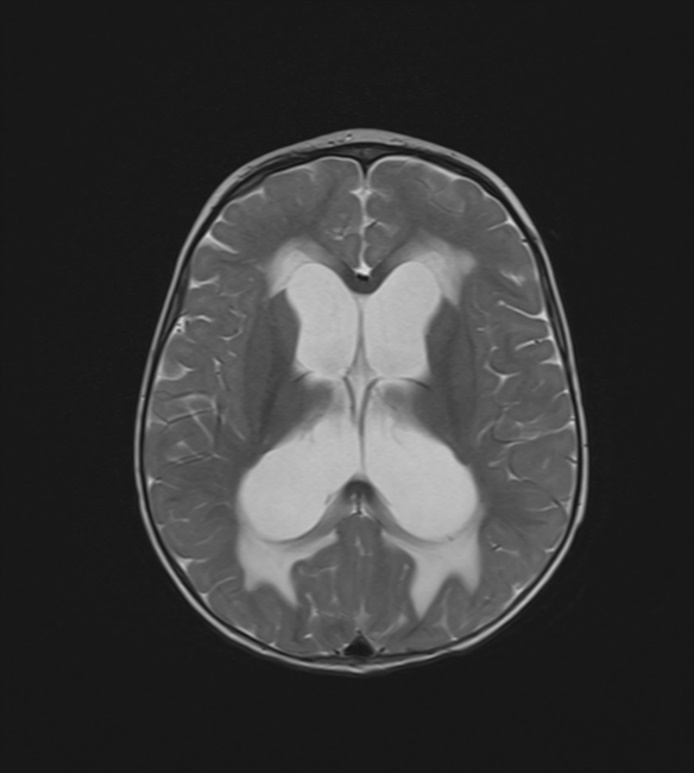


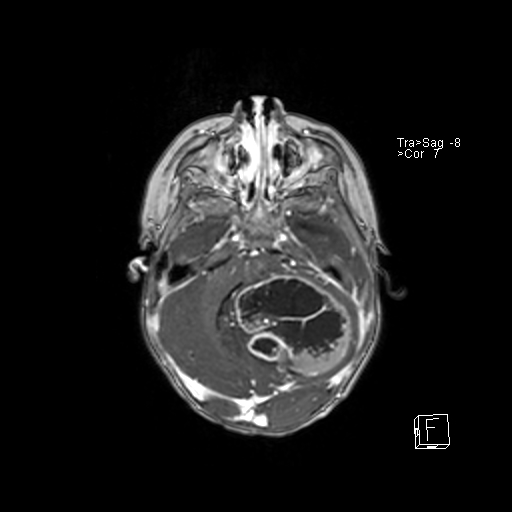

Supplement: Supplementary file 1 — Fig S1 [file CCR3-9-1472-s001.docx]
