# Supplementary material for: NTRK2 Fusion driven pediatric glioblastoma: Identification of oncogenic Drivers via integrative Genome and transcriptome profiling
Source: Clin Case Rep. 2021 Feb 10;9(3):1472–7. doi: 10.1002/ccr3.3804 (PMC7981675; doi:10.1002/ccr3.3804)
Supplement: Supplementary file 2 — Supplementary Material [file CCR3-9-1472-s002.docx]

**SUPPLEMENTAL INFORMATION**

**Ethics, privacy and consent:**

Written, informed consent for sequencing and publication of clinical and genomic data was obtained for each patient in the program, and all protocols and procedures in the program were approved by the University of British Columbia Research Ethics Committee (REB No. H12-00137) and represents part of the Personalized Oncogenomics trial NCT02155621 <https://clinicaltrials.gov/ct2/show/study/NCT02155621>. Raw sequencing data was maintained within a secure computing environment at the Michael Smith Genome Sciences Centre, and clinical data was maintained by physicians and a dedicated research team at the BC Cancer Agency.

**METHODS**

**Pathology / Histology / Immunohistochemistry:**

For light microscopy, formalin fixed and paraffin embedded (FFPE) blocks were sectioned at 6 μm for hematoxylin and eosin (H&E) staining and 4 μm for immunohistochemistry. All microscopic slides were prepared via standard automated techniques, including special staining for reticulin (Gordon and Sweet’s method) and periodic acid Schiff with diastase. Most immunohistochemical slides were prepared via the Ventana BenchMark XT Autostainer (Ventana Medical Systems Inc., Tucson, AZ, USA) and the biotin based Ventana iVIEW Universal DAB Detection Kit. Primary antibodies for immunohistochemistry included (vendor, dilution): glial fibrillary acidic protein, GFAP [Cell Marque, ready to use (RTU) prediluted solution]; Ki67 (Ventana, RTU); synaptophysin (Ventana, RTU); epithelial membrane antigen, EMA (Ventana, RTU); NeuN (Chemicon, 1: 400); BAF47/INI1 (BD Bioscience, 1:100); p53 (Ventana, RTU); phosphorylated neurofilament protein, pNFP (Cedarlane Covance, 1:5000); CD68 (Ventana, RTU); LCA (Ventana, RTU); ATRX (Sigma, 1:200); IDH1 (Dianova; 1:100).

Immunohistochemistry for PD-L1 was performed using primary clone SP142 and the method were appropriately validated prior to this study and described in detail previously.^1^ Briefly, sections were deparaffinized then subjected to antigen retrieval using Biocare’s Diva decloaking solution and decloaking chamber (Biocare, Concord, USA).  They were then loaded onto an Intellipath FLX autostainer, blocked with peroxidased-1 and background sniper and then incubated with PD-L1 clone SP142 (Spring Bioscience, Pleasanton, USA) at 1/100 dilution in Da Vinci Green diluent at room temperature for 30 minutes.  Slides were then washed and incubated with Mach2 Rabbit-HRP polymer for 30 minutes at room temperature and detected with IP DAB chromogen for 5 minutes.  Nuclei were counterstained with a 1/10 dilution of CAT hematoxylin then slides were again washed, air dried and coverslipped with Ecomount.

**Personalized Oncogenomics:**

Whole-genome transcriptome analysis was performed on the tissue obtained from resection of the residual tumor within the left posterior fossa, using methods as previously described by our group.^2^ The sample was frozen and embedded in optimal cutting temperature compound for DNA and RNA extractions, as well as frozen section for histologic correlation. Matching normal DNA was extracted from peripheral blood leukocytes.

Sequencing:

Paired-end DNA and RNA libraries were generated by a specialized team at Canada’s Michael Smith Genome Sciences Centre (GSC), and sequencing was performed using the Illumina HiSeq platform v3 (Illumina, San Diego, California).

Bioinformatic analysis:

Reads were aligned to the human genome (GRCh37-lite) using BWA (0.5.7). Reads from multiple lanes were merged and duplicate marked using Picard (v1.38). Variants were called using mpileup (SAMtools v0.1.17) and a subsequently filtered with varFilter. Each tumour sample (metastasis and primary) was compared to the normal sample to identify somatic copy number variants (CNAseq v0.0.6), LOH events (APOLLOH v0.1.1), single nucleotide variants (SAMtools v0.1.17, MutationSeq v1.0.2), and small insertions and deletions (Strelka v0.4.6.2).

RNA-Seq reads were analyzed with Jaguar to include alignments to a database of exon junction sequences and subsequent repositioning onto the genomic reference. RNA-Seq data was processed using the WTSS pipeline coverage analysis (v1.1) to determine gene and exon read counts and normalized expression level. Expressed variants were called with SNVMix2 (v0.12.1-rc1) and SAMtools (v0.1.13). Gene expression in the tumour was compared to a compendium of normal tissues and to one or more normal libraries from the same tissue type to identify up- and down-regulated genes. Both genomic and RNA-Seq tumour data were also assembled using Trans-ABySS (v1.4.3) to identify structural variants and fusion genes.

Variants were annotated to genes using the Ensembl database (v59, 68). Genes were linked to cancer pathways using COSMIC, KEGG, and Ingenuity Pathway Analysis, and linked to drugs using DrugBank and the Therapeutic Target Database. Literature review for drug/target combinations and pharmacogenetics was integrated to identify potential therapeutic recommendations.

**REFERENCES**

1. Sheffield, B.S.*, et al.* Investigation of PD-L1 Biomarker Testing Methods for PD-1 Axis Inhibition in Non-squamous Non-small Cell Lung Cancer. *J Histochem Cytochem* **64**, 587-600 (2016).

2. Jones, M.R.*, et al.* Successful targeting of the NRG1 pathway indicates novel treatment strategy for metastatic cancer. *Ann Oncol* **28**, 3092-3097 (2017).
